# ThermoScan: Semi-automatic Identification of Protein Stability Data From PubMed

**DOI:** 10.3389/fmolb.2021.620475

**Published:** 2021-03-25

**Authors:** Paola Turina, Piero Fariselli, Emidio Capriotti

**Affiliations:** ^1^ Department of Pharmacy and Biotechnology (FaBiT), University of Bologna, Bologna, Italy; ^2^ Department of Medical Sciences, University of Torino, Torino, Italy

**Keywords:** protein stability, text mining, document classification, automated literature mining, thermodynamic data

## Abstract

During the last years, the increasing number of DNA sequencing and protein mutagenesis studies has generated a large amount of variation data published in the biomedical literature. The collection of such data has been essential for the development and assessment of tools predicting the impact of protein variants at functional and structural levels. Nevertheless, the collection of manually curated data from literature is a highly time consuming and costly process that requires domain experts. In particular, the development of methods for predicting the effect of amino acid variants on protein stability relies on the thermodynamic data extracted from literature. In the past, such data were deposited in the ProTherm database, which however is no longer maintained since 2013. For facilitating the collection of protein thermodynamic data from literature, we developed the semi-automatic tool ThermoScan. ThermoScan is a text mining approach for the identification of relevant thermodynamic data on protein stability from full-text articles. The method relies on a regular expression searching for groups of words, including the most common conceptual words appearing in experimental studies on protein stability, several thermodynamic variables, and their units of measure. ThermoScan analyzes full-text articles from the PubMed Central Open Access subset and calculates an empiric score that allows the identification of manuscripts reporting thermodynamic data on protein stability. The method was optimized on a set of publications included in the ProTherm database, and tested on a new curated set of articles, manually selected for presence of thermodynamic data. The results show that ThermoScan returns accurate predictions and outperforms recently developed text-mining algorithms based on the analysis of publication abstracts.

**Availability:** The ThermoScan server is freely accessible online at https://folding.biofold.org/thermoscan. The ThermoScan python code and the Google Chrome extension for submitting visualized PMC web pages to the ThermoScan server are available at https://github.com/biofold/ThermoScan.

## Introduction

A key aspect for characterizing the relationship between genotype and phenotype is the study of the impact of amino acid variants on protein function and structure (Thusberg and Vihinen, 2009; Compiani and Capriotti, 2013). To address this task, several tools for predicting the effect of variants on protein stability have been developed (Sanavia et al., 2020). The implementation of these methods requires a large and accurate set of experimental data, both for training and benchmarking. Although many protein folding databases were developed in the past (Bava et al., 2004; Fulton et al., 2007; Wagaman et al., 2014; Pancsa et al., 2016; Manavalan et al., 2019) some of them were discontinued or no longer maintained (Bava et al., 2004; Fulton et al., 2007). Among them, ProTherm (Kumar et al., 2006), the most comprehensive resource for thermodynamic data on protein variants, was not updated since 2013, and its maintenance was discontinued. Therefore, the need for curated databases on the thermodynamics and kinetics of protein folding has become urgent for implementation of accurate prediction methods.

In general, the collection of data from scientific literature is an expensive and time-consuming process requiring careful selection of keywords and queries for web searching (Fleuren and Alkema, 2015). As a consequence, during the last decades, several text-mining tools have been developed to speed up the data collection process (Rebholz-Schuhmann et al., 2012). Given the complexity and large variety of biological data, such searching tools were customized to address specific tasks (Huang and Lu, 2016). In particular, different approaches have been developed for identifying protein-protein interactions (Krallinger et al., 2008), drug-drug interactions (Zeng et al., 2019) and drug-phenotype relationships (Garten and Altman, 2009). Other methods identify gene functions (Soldatos et al., 2015) and define the role of molecules involved in biological processes (Wang et al., 2011). Currently, text-mining tools are used in daily life science research activity to improve web search (Ananiadou et al., 2010) and facilitate the database curation process (Yeh et al., 2003; Wei et al., 2012; Karp, 2016).

In this context, we developed ThermoScan, a new method for facilitating the collection and curation of thermodynamic data. Aiming at maximizing the extent of automatic vs. manual curation, ThermoScan is based on a semi-automatic text-mining algorithm for identifying experimental data on protein stability within the publicly accessible literature. ThermoScan reads the Open Access full-text manuscripts, ranks them according to the likelihood of finding the experimental thermodynamic data, and extracts relevant parts of the manuscript from paragraphs and tabular items. In addition, we evaluated the performance of ThermoScan in the detection of thermodynamic data in comparison with two existing web-server tools for documents classification (Fontaine et al., 2009; Simon et al., 2019).

## Methods

ThermoScan is a semi-automatic method for retrieving protein thermodynamic data from literature. The method scans the PubMed Central full-text HTML page and calculates a score for identifying manuscripts reporting experimental protein thermodynamic data in paragraphs and tables.

### Datasets

For optimizing and testing the performance of ThermoScan we collected different datasets of articles reporting protein thermodynamic data (positives) or not (negatives). The initial set of positives (Pos-PT) was collected by considering 157 Open Access PMC articles referenced in the ProTherm database. Two negative sets of publications were selected from the PMC Open Access repository using different searching keywords. In detail we considered only the full-text articles available in HTML format and containing the terms “*protein*” and “*stability*” (Neg-PS) or “*protein*” and “*unfolding*” (Neg-PU). For the Neg-PS dataset we restricted the search to the first 2,000 articles. Thus, the Neg-PS and Neg-PU negative sets, obtained by restricting the literature search to the period 2000–2010, were composed of 2,000 and 583 manuscripts respectively.

For testing the performance of ThermoScan, we selected a set of 296 recently published (2011–2019) Open Access PMC articles with a PubMed search of the keywords “*protein*,” “*stability*” and “*unfolding*”. The manual curation of these articles, based on stringent criteria, allowed the identification of 194 manuscripts reporting experimental protein folding data. The remaining 102 papers, initially retained as negatives, were filtered excluding 37 articles reporting only protein thermodynamic data from binding or *in silico* experiments. With this manual procedure, we generated the New-PSU dataset, composed of 194 positive and 102 negative articles, and the Snew-PSU, composed of the same number of positives and 65 high-quality negatives. The composition of the datasets is summarized in Supplementary Table S1. The PMCIDs of the manuscripts collected in all the datasets are available as Supplementary File.

### Manuscript Processing and Word Selection

Full-text articles in HTML format are parsed using the BeautifulSoup *Python* library (https://www.crummy.com/software/BeautifulSoup/). BeautifulSoup is used for extracting the text between paragraphs (*<p>*) and tables (*<table>*) tags. After extraction of the text included in the paragraphs and tables of each manuscript, the Natural Language Toolkit (NLTK) platform (https://www.nltk.org/) (Bird et al., 2009) is used for removing stopwords and for the lemmatization process. In particular, we use the *WordNetLemmatizer* function of NLTK for determining the word’s lemma. After processing the manuscript with NLTK, the text is analyzed for identifying the words associated with protein thermodynamic concepts. In detail, we compared the frequency of the words in the manuscript of Pos-PT dataset against the Neg-PS dataset using a binomial distribution. The words were ranked on the basis of the *p*-value obtained from the complementary cumulative binomial distribution. Such *p*-value represents the probability of observing, in the Pos-PT dataset, a number of manuscripts with a given word higher than expected from the background probability, as estimated in the Neg-PS dataset. According to the *p*-values, calculated using the binomial survival function of the binomial distribution (Supplementary Table S2), the 5 words with lowest score were: unfolding, two-state, denaturant, dichroism and midpoint.

### Text Mining and Scoring

ThermoScan processes the full-text article in HTML searching for significant protein thermodynamic words grouped in four classes:

•**Thermodynamic concepts (TC):** Important words frequently appearing in protein thermodynamic studies (unfolding, two-state, denaturant, dichroism, midpoint).
•**Thermodynamic variables (TV)** Words are identified by a regular expression matching the abbreviations of the main thermodynamic variables (ΔG, ΔH, ΔTm, etc.).
•**Units of measure (UM):** Words are identified by a regular expression matching the main units of measure used in thermodynamic experiments (kcal/mol, kJ/mol, etc)
•**Computational concepts (CC):** Words referring to computational studies (simulation, molecular dynamics, force field, predict, etc.).

The text extracted from the manuscript is searched for the 5 words in the first group. If one of the words is found, all the significant terms are extracted using each of the four regular expressions representing the four classes. The codes of the four regular expressions are reported in Supplementary Materials.

For each article, ThermoScan calculates an empirical score based on the four classes of words defined above. Our approach returns the total and the single paragraph/table scores. A positive partial score is assigned to the items matching the first three classes (thermodynamic concepts, thermodynamic variables and units of measures), and a negative one to the items matching the fourth class (computational concepts).

The paragraph/table score is calculated by summing the scores of the individual matches without repetitions. The individual scores of the different classes of words are the following:

•two-state = unfolding = denaturant = midpoint = dichroism = 1
•Cp = Tm = 1, ΔX = 2, ΔΔX = 3 (X = Cp, Tm, UG, GU, G, H, T, U)
•°C = 1, E/C = 2 (E = kcal, kJ; C = mol, mole, mole/°C, mol/°C, mol/K, mol/M)
•simulation = molecular dynamics = force field = charmm = gromacs = amber = PBSA = GBSA = predict = −1; md simulation = −2

The total score assigned to the article is obtained by summing all paragraph/table scores. For the classification task, we considered two alternative measures, corresponding to the maximum (Max) or to the average (Mean) paragraph/table score for each paper.

Although not used at this stage for the classification task, ThermoScan additionally searches for thermodynamic data relative to binding processes, considering the following terms: binding, affinity, dissociation, interaction, ppi, protein-protein, kcat/Km.

### Method optimization and Testing

For optimizing the performance of ThermoScan we maximized the performance of a binary classifier discriminating between manuscripts reporting protein thermodynamic data and not. In general, this task can have different difficulty levels depending on the selection of the negative set. To select a fair negative set of manuscripts, we considered those collected in the Neg-PS and Neg-PU datasets, which include the terms “*protein*” and “*stability*,” or “*protein*” and “*unfolding*,” respectively. From Neg-PS and Neg-PU datasets we generated 10 randomly selected sets of 157 negative manuscripts in equal proportion, to be compared with those collected in the Pos-PT dataset. With this procedure we generated 10 training sets that only differ by the subset of negatives. Using the procedure described above, for each manuscript we calculated the maximum (Max) and average (Mean) scores of the extracted paragraphs and tables. In addition, we evaluated the relative contributions of the three main groups of words (thermodynamic concepts, thermodynamic variables and units of measures) to the prediction power of ThermoScan by calculating the performance achieved when using different groups combinations. In particular we evaluated the performance of three alternative methods considering:

•thermodynamic concepts alone (TC);
•thermodynamic variables and units of measures (TV ∪ UM);
•thermodynamic concepts, thermodynamic variables and units of measures (TC ∪ TV ∪ UM).

The results obtained with the three combinations were compared with those obtained by including all four groups of words defined above.

For ThermoScan optimization we selected the classification thresholds that maximized the Matthews Correlation Coefficient (see Methods section in Supplementary Materials), and finally we tested the ThermoScan performance on the two testing sets (New-PSU, Snew-PSU) by applying the same classification thresholds.

The performance of ThermoScan was then compared with those achieved by MedlineRanker (Fontaine et al., 2009) and BioReader (Simon et al., 2019). The performances of the two text mining methods (MedlineRanker and BioReader), which are both based on the analysis of the manuscript abstract, were evaluated on the New-PSU, Snew-PSU datasets. All the performance measures are defined in Supplementary Materials.

## Results

Here we present the results achieved by ThermoScan in the selection of manuscripts reporting experimental protein thermodynamic data from PubMed. We first optimized ThermoScan in a training step, then tested its performance on a blind set of manually curated articles, and finally compared such performance with those achieved by MedlineRanker (Fontaine et al., 2009) and BioReader (Simon et al., 2019).

### ThermoScan Optimization

**FIGURE 1:**
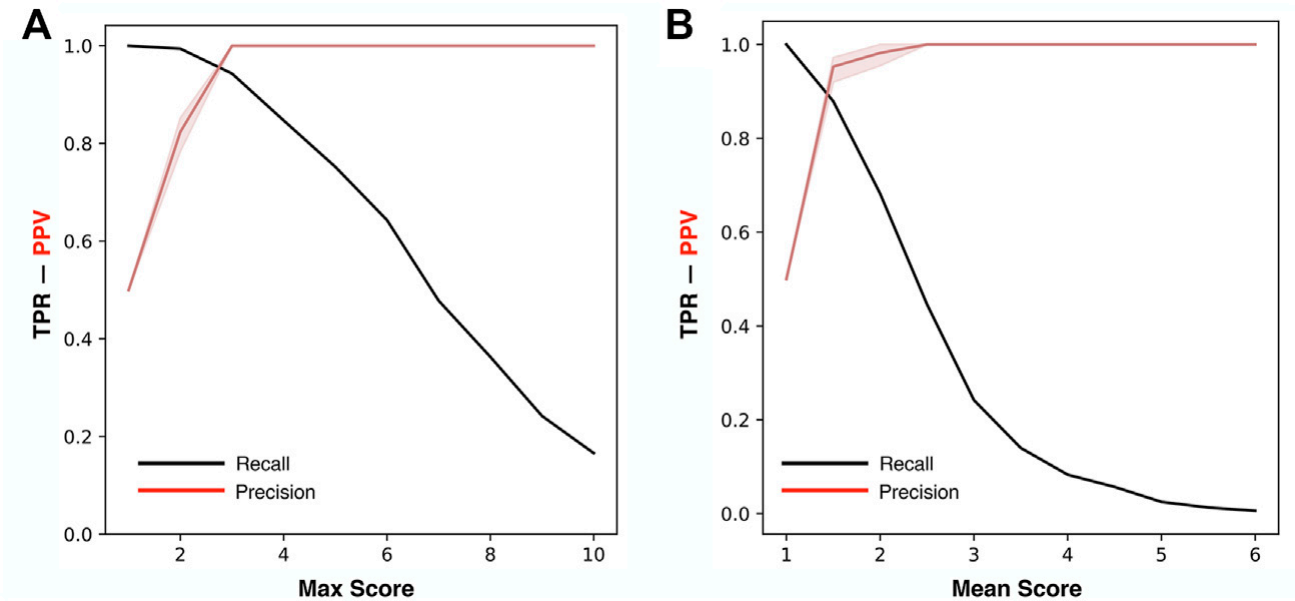
Precision and Recall of ThermoScan at different classification thresholds. The plots show the performance based on the Max **(A)** and Mean **(B)** scores. The performance measures TPR (black) and PPV (red) are defined in Supplementary Materials. The shaded area represents the range between the minimum and maximum scoring values.

**FIGURE 2:**
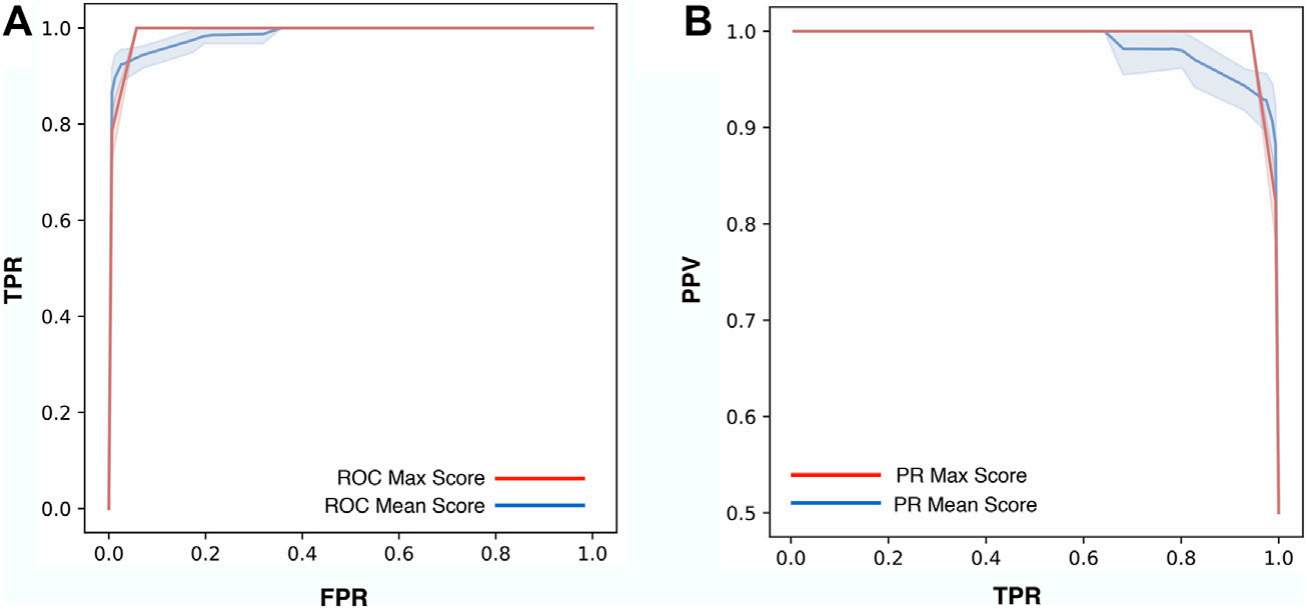
Performance measures of ThermoScan based on the Max (red) and Mean (blue) scores. The plots show the AUC (Area Under the receiving operating characteristic Curve). The shaded area represents the range between the minimum and maximum scoring values **(A)** and the AUPR (Area Under the Precision-Recall curve) **(B)** for the two scoring systems. The TPR, FPR, and PPV performance measures are defined in Supplementary Materials.

For the optimization of ThermoScan we calculated its performance considering both the maximum (Max) and the average (Mean) scores assigned to each part (paragraph/table) of the manuscript. The performance of ThermoScan was calculated using a positive set of 157 manuscripts from Protherm containing protein thermodynamic data (Pos-PT) and a negative set with an equal number of articles not containing any thermodynamic information (randomly selected from Neg-PU and Neg-PS datasets, described in the Methods section). All the performance measures (defined in the Supplementary Materials) were averaged over 10 random samplings of the negative subset. The detailed results obtained with both Max and Mean scoring systems are reported in Supplementary Tables S3, S4; Table 1 summarizes the optimal performance measures from Supplementary Tables S3, S4 for both the Max and Mean scoring systems. In detail, the method based on the maximum score achieved 3% higher accuracy (Q_2_) and 5% higher Matthews correlation coefficient (MCC). In Figure 1, the Precision (PPV) and Recall (TPR) values from Supplementary Tables S3, S4 are plotted as a function of Max (Figure 1A) and Mean (Figure 1B) scoring threshold. The results show that the best performance was achieved with the Max scoring system with threshold ≥3. Alternative scores of the performance are based on the AUC (Area Under the receiving operating characteristic Curve) and on the AUPR (Area Under the Precision-Recall curve) which are shown in Figure 2. Also, these results confirm that the Max scoring system achieved the best performance.

**TABLE 1:** Optimized performance of ThermoScan based on the maximum (Max) and average (Mean) scores. The performance measures are defined in Supplementary Materials. The standard deviation of all the performance measures are ≤0.01.

Score	TH	Q_2_	TNR	NPV	TPR	PPV	MCC	F1	AUC	AUPR
Max	3.00	0.97	1.00	0.95	0.94	1.00	0.94	0.97	0.99	0.99
Mean	1.36	0.94	0.94	0.95	0.95	0.94	0.89	0.94	0.98	0.99

In summary, the above analysis shows that the binary classifier results in a higher performance when based on the maximum paragraph/table score rather than on the average score.

### ThermoScan Testing and Benchmarking

ThermoScan was tested calculating its performance on two sets (New-PSU and Snew-PSU) obtained by searching in the Open Access PMC articles having the words “*protein*,” “*stability*” and “*unfolding*” in their abstracts. The classification was performed using the same threshold values obtained in the optimization steps. The results reported in Table 2 show that ThermoScan achieved the highest performance on the testing set Snew-PSU, obtained by removing 37 manuscripts of difficult classification, (i.e. reporting protein thermodynamic data from binding or *in silico* experiments only). Indeed, when comparing the performances of both versions of ThermoScan (Max and Mean) on the Snew-PSU and New-PSU datasets, the method results in ∼10% better accuracy and 20% better Matthews correlation coefficient on the first one. The version of ThermoScan based on the maximum paragraph/table score achieved an overall accuracy of 91% and a Matthews correlation coefficient of 0.76. These results are the most similar ones to those reached in the optimization step. Furthermore, to estimate the filtering capabilities of ThermoScan, we analyzed a set of ∼700,000 manuscripts from the PubMed Central FTP website (https://ftp.ncbi.nlm.nih.gov/pub/pmc/manuscript/), which required on average ∼4 s for each article. By using a scoring threshold of 6, ThermoScan selects ∼2,200 items (0.3%), which, according to our analysis of the New-PSU testing set, are expected to include less than 4% of false positives. Finally, we compared the performance of ThermoScan with those of MedlineRanker (Fontaine et al., 2009) and BioReader (Simon et al., 2019) which are based on the analysis of the manuscript abstracts. As shown in Table 3, ThermoScan, that analyzes the full-text manuscript, results in better performance than MedlineRanker and BioReader on both New-PSU and Snew-PSU datasets. In almost all cases ThermoScan reached ∼15% higher overall accuracy and ∼30% higher Matthews correlation coefficient with respect to MedlineRanker and BioReader. Given the different amount of information in input, the performance of ThermoScan can not be directly compared with those of MedlineRanker and BioReader. Our analysis shows that full-text classification-based methods do tend to have higher discriminating power than methods based on the analysis of the abstract, even though the latter can deal with larger sets of articles in a shorter amount of time.

**TABLE 2:** Performance of ThermoScan on the New-PSU and Snew-PSU datasets. The ThermoScan thresholds obtained in the optimization step with maximum and mean paragraph/table scoring methods are 3.00 and 1.36 respectively. The performance measures are defined in Supplementary Materials.

Score	Dataset	Q_2_	TNR	NPV	TPR	PPV	MCC	F1	AUC	AUPR
Max	New-PSU	0.80	0.49	0.88	0.96	0.78	0.55	0.86	0.86	0.86
	Snew-PSU	0.91	0.75	0.88	0.96	0.92	0.76	0.94	0.96	0.94
Mean	New-PSU	0.80	0.59	0.77	0.91	0.81	0.53	0.85	0.83	0.82
	Snew-PSU	0.89	0.83	0.75	0.91	0.94	0.71	0.92	0.92	0.91

**TABLE 3:** Comparison of the performance of ThermoScan (based on maximum paragraph/table score) with BioReader and MedlineRanker on the New-PSU and Snew-PSU datasets. The classification thresholds for BioReader and MedlineRanker and ThermoScan are 0.022, 0.027 and three respectively. The performance measures are defined in Supplementary Materials.

Method	Dataset	Q_2_	TNR	NPV	TPR	PPV	MCC	F1	AUC	AUPR
BioReader	New-PSU	0.66	0.59	0.50	0.70	0.76	0.28	0.73	0.64	0.72
	Snew-PSU	0.70	0.69	0.43	0.70	0.87	0.34	0.77	0.69	0.75
MedlineRanker	New-PSU	0.63	0.63	0.47	0.63	0.76	0.25	0.69	0.70	0.67
	Snew-PSU	0.70	0.68	0.43	0.70	0.87	0.34	0.78	0.78	0.72
ThermoScan	New-PSU	0.80	0.49	0.88	0.96	0.78	0.55	0.86	0.86	0.86
	Snew-PSU	0.91	0.75	0.88	0.96	0.92	0.76	0.94	0.96	0.94

### Contribution to Performance

To evaluate the contribution to the performance of ThermoScan of each group of words included in the manuscript processing, we assessed the performance of three alternative methods considering a subset of groups (see *Method optimization and testing* paragraph in the Methods section). In particular, we compared the performance of ThermoScan with the three following approaches based on:

i.the thermodynamic concepts alone (TC);
ii.the thermodynamic variables and units of measure (TV ∪ UM);
iii.all previous groups (TC ∪ TV ∪ UM).

On the training sets (Pos-PT, Neg-PS and Neg-PU), the results of the comparison between ThermoScan, which includes four groups of words (TC ∪ TV ∪ UM ∪ CC), and the alternative methods described above are reported in Supplementary Tables S5, S6. This analysis shows that the predominant contribution to the classification power is given by the 5 words belonging to the group of the thermodynamic concepts. We also noticed that the combination, which significantly contributes to improve the performance, includes all three groups: both the thermodynamic concepts and variables, together with the units of measure. Indeed, considering the classifier based on the maximum paragraph/table score, the method based on the combination of the three groups of words results in 4% better overall accuracy and 7% better Matthews correlation coefficient with respect to the methods based on thermodynamic concepts alone (Supplementary Table S5). Although no significant improvement of the performance is resulting from adding the computational concepts (CC), this negative score, which is included in ThermoScan, is important for penalizing the manuscripts reporting in silico protein stability data. A similar improvement is observed on the testing sets New-PSU and Snew-PSU (Supplementary Tables S7–S10). In the testing step we observed an improvement of NPV (negative predicted value) and TNR (true negative rate) of 2 and 4% respectively when comparing ThermoScan with the method based on the three groups of words (TC ∪ TV ∪ UM).

### Identification of In-Silico Data and Manuscripts

Identifying in-silico articles, which represented less than 10% of our testing set, remains a critical issue, especially when the article texts include reference to, and description of, experimental data. To penalize articles presenting in-silico data only, we defined a negative score based on the presence of the computational concepts (CC). The maximum penalization score for a paragraph is -2 when the words “md simulation” is found. Although the addition of the CC does not significantly improve the performance of the automatic evaluation, it can help during the manual curation process to detect and discard possible false positives.

### ThermoScan Web Server and Code

We developed a web server version of ThermoScan that takes in input a list of manuscript identifiers (PMCID, PMID or DOI) and returns a table with the scores associated with each article. Each identifier in the output is linked to a webpage showing significant paragraphs and tables which include protein thermodynamic terms. Words belonging to the main three classes defined in the Method section (thermodynamic concepts, thermodynamic variables, units of measure) are highlighted in *red*. To facilitate the curation process and avoid the selection of in-silico data, the output of the webserver displays the CC terms in blue and returns a score related to their presence. For better help in identifying the possible presence of thermodynamic data on protein mutants, the potential amino acid variants are highlighted in *green*. For each manuscript, the server calculates the total score and the maximum score for the extracted paragraphs and tables. An example of the ThermoScan server output is available at the page https://shorturl.at/cetwG. To analyze the HTML pages of manuscripts with restricted access, we developed a GoogleChrome app that allows the user to submit the content of a web page, visualized on the user’s browser, directly to the ThermoScan server. Furthermore, the ThermoScan python script for the local scanning of the PMC articles is made available through GitHub.

## Discussion

In this paper we present ThermoScan, a text-mining algorithm for the selection and fine-grained classification of Open Access PMC articles, aimed at retrieving literature data on the thermodynamic stability of proteins and their variants. Although the direct comparison of the performance of methods with different input features is not straightforward, our results show that ThermoScan, which is based on the analysis of full-text articles, outperforms existing web services based on the analysis of the manuscript abstracts (Fontaine et al., 2009; Simon et al., 2019), thus constituting a new valuable tool to semi-automatically collect protein thermodynamic data. Furthermore, the web interface, which displays relevant parts of the article, makes ThermoScan a valuable complementing tool for refining the search of protein thermodynamic data. In conclusion, our method achieves a high discrimination power by analyzing full-text articles, by fine-tuning the classification thresholds, and by using a tailored subset of specific symbols and words. Given the trend toward an increasing amount of in-silico only studies in the literature repositories, in the future more sophisticated search strategies should be implemented, to avoid the selection of manuscripts reporting in-silico data only, which contribute to increasing the rate of false positives. Nevertheless we expect that ThermoScan will significantly support and accelerate the updating and curation of new databases for collection of protein thermodynamic data. Such data are essential for characterizing the relationship between protein sequence and structure and for the development of more accurate methods for predicting the impact of amino acid variants on protein stability.

## Data Availability

The original contributions presented in the study are included in the article/Supplementary Material, further inquiries can be directed to the corresponding authors.

## Abbreviations

AUC, Area Under the receiving operating characteristic Curve; AUPR, Area Under the Precision-Recall curve; CC, Computational Concepts; F1 score, harmonic mean of PPV and TPR; MCC, Matthews correlation coefficient; NPV, Negative Predicted Values; Q 2, overall accuracy; PMC, PubMed Central; PPV, Positive Predicted Values; TC, Thermodynamic Concepts; TPR, True Positive Rate; TNR, True Negative Rate; TV, Thermodynamic Variables; UM, Units of Measure.
