# Supplementary material for: ThermoScan: Semi-automatic Identification of Protein Stability Data From PubMed
Source: Front Mol Biosci. 2021 Mar 25;8:620475. doi: 10.3389/fmolb.2021.620475 (PMC8027235; doi:10.3389/fmolb.2021.620475)
Supplement: Supplementary file 2 [file presentation1.pdf]

SUPPLEMENTARY MATERIALS

ThermoScan: Semi-automatic identification of protein stability data from PubMed.

Paola Turina [1], Piero Fariselli [2], and Emidio Capriotti [1*]*

[1] Department of Pharmacy and Biotechnology (FaBiT), University of Bologna, Via F. Selmi 3, 40126 Bologna, Italy.

[2] Department of Medical Sciences, University of Torino, Via Santena 19, 10126, Torino, Italy.

Contacts: piero.fariselli@unito.it and emidio.capriotti@unibo.it

Methods

Text mining regular expression

The ThermoScan algorithm is based on the search of 4 classes of words: thermodynamic concepts (TC), thermodynamic variables (TV), units of measure (UM) and computational concepts (CC). For searching each class of words the following regular expressions are used.

- **TC**: u'(?:\W|^)(two-state|unfolding|denaturant|midpoint|dichroism)'
- **TV**: u'(?:\W|^)((?:(?:\u2206|\u0394){1,2}(?:Cp|Tm|UG|GU|G|H|T))|(?:Cp|Tm)
- **UM**:u'(?:(?:(?:kcal|kj)(?:\/mole?(?:\/[\u00b0|\u00b4]C)?|[\s*\.\u00b7\u22c5]?(?:mole?[\-\u2212]1)|\/M\/mol|\/\(mol\s[MK]\)|\s[MK][\-\u2212]1)?)|(?:[\u00b0|\u00b4]C))'
- **CC**: u'(?:\W|^)(md simulation|simulation|molecular dynamics|force field|charmm|gromacs|amber|PBSA|GBSA|predict)'

Measures of performance

ThermoScan is a binary classifier that discriminates between manuscripts reporting experimental data on the thermodynamics of protein folding (*positive manuscripts*) and manuscripts that do not (*negative manuscripts*). The binary classification (positive/negative manuscripts) is made both on the basis of the maximum (Max) and the average (Mean) scores assigned to each paragraph/table of the manuscript. Thus, if the Mean/Max score is greater or equal than the selected threshold, the manuscript is predicted to be *positive*.

In all the performance measures - assuming that *positives manuscripts* include protein thermodynamic data and that *negative manuscripts* do not - TP (true positives) are correctly predicted manuscripts including protein thermodynamic data, TN (true negatives) are correctly predicted manuscripts not reporting protein thermodynamic data, FP (false positives) and FN (false negatives) are incorrectly predicted manuscripts.

The predictor performance was evaluated using the following metrics: true positive and negative rates (*TPR, TNR*), positive and negative predicted values (*PPV, NPV*), *F1* score and overall accuracy (Q_2)

$$Manuscripts\ with\ Protein\ Thermodynamic\ Data: \quad PPV = \frac{TP}{TP+FP} \quad TPR = \frac{TP}{TP+FN}$$

$$Manuscripts\ without\ Protein\ Thermodynamic\ Data: NPV = \frac{TN}{TN+FN} \quad TNR = \frac{TN}{TN+FP} \qquad \text{[Eq. 1]}$$

$$F1 = \frac{2TP}{2TP+FP+FN} \quad Q_2 = \frac{TP+TN}{TP+FP+TN+FN}$$

We computed the Matthews correlation coefficient *MCC* (Eq. 2) as:

$$MCC = \frac{TP \times TN - FP \times FN}{\sqrt{(TP+FP)(TP+FN)(TN+FP)(TN+FN)}} \qquad \text{[Eq. 2]}$$

We also calculated the AUC (Area Under the receiver operating characteristic Curve), by plotting TPR as a function of FPR at different score thresholds. With a similar procedure we calculated the AUPR (Area Under the Precision-Recall curve) where the Precision and Recall are equal to PPV and TPR respectively (see Eq. 1).

Tables

Dataset	Filter	Manuscripts	Positives	Negatives
Pos-PT	Open Access PMC in HTML format	157	157	0
Neg-PS*	Open Access PMC in HTML format<br>Terms: Protein+Stability<br>Period: 2000-2010	2,000	0	2,000
Neg-PU	Open Access PMC in HTML format<br>Terms: Protein+Unfolding<br>Period: 2000-2010	583	0	583
New-PSU	Open Access PMC in HTML format<br>Terms: Protein+Stability+Unfolding<br>Period: 2011-2018	296	194	102
Snew-PSU	Open Access PMC in HTML format<br>Terms: Protein+Stability+Unfolding<br>Period: 2011-2018<br>NOT (protein binding OR *in silico*)	256	194	65

Table S1. Composition of datasets used for optimization and testing of ThermoScan.

* The first 2,000 manuscripts were selected.

WORDS	$P_{Neg\text{-}PS}$	$N_{Pos\text{-}PT}$	p-value
unfolding	2.50E-02	149	0.00E+00
two-state	9.50E-03	113	1.50E-192
denaturant	4.50E-03	95	9.10E-182
dichroism	1.45E-02	100	1.50E-143
midpoint	1.75E-02	94	1.50E-123
thermodynamic	3.85E-02	107	3.70E-113
kcal	2.75E-02	98	3.40E-112
ΔG	6.00E-03	68	1.10E-108
ellipticity	4.00E-03	62	3.20E-107
enthalpy	2.00E-03	51	4.90E-99

Table S2. Top ranking protein thermodynamics words obtained comparing the occurrence of the words in Pos-PT and Neg-PS.

TH	Q2	TNR	NPV	TPR	PPV	MCC	F1
1	0.50	0.00	0.00	1.00	0.50	0.00	0.67
2	0.89	0.79	0.99	0.99	0.82	0.80	0.90
3	0.97	1.00	0.95	0.94	1.00	0.94	0.97
4	0.92	1.00	0.87	0.85	1.00	0.86	0.92
5	0.88	1.00	0.80	0.75	1.00	0.78	0.86
6	0.82	1.00	0.74	0.64	1.00	0.69	0.78
7	0.74	1.00	0.66	0.48	1.00	0.56	0.65
8	0.68	1.00	0.61	0.36	1.00	0.47	0.53
9	0.62	1.00	0.57	0.24	1.00	0.37	0.39
10	0.58	1.00	0.55	0.17	1.00	0.30	0.28
**3**	**0.97**	**1.00**	**0.95**	**0.94**	**1.00**	**0.94**	**0.97**

Table S3. Performance of ThermoScan based on the different thresholds (TH) of maximum paragraph/table scores, ranging from 1 to 10. The performance measures are defined in the Methods section. The standard deviations of all performance measures are ≤0.03.

TH	Q2	TNR	NPV	TPR	PPV	MCC	F1
1.00	0.50	0.00	0.00	1.00	0.50	0.00	0.67
1.50	0.92	0.96	0.89	0.88	0.95	0.84	0.91
2.00	0.83	0.99	0.76	0.68	0.98	0.70	0.80
2.50	0.72	1.00	0.64	0.45	1.00	0.54	0.62
3.00	0.62	1.00	0.57	0.24	1.00	0.37	0.39
3.50	0.57	1.00	0.54	0.14	1.00	0.27	0.25
4.00	0.54	1.00	0.52	0.08	1.00	0.21	0.15
4.50	0.53	1.00	0.52	0.06	1.00	0.17	0.11
5.00	0.51	1.00	0.51	0.03	1.00	0.11	0.05
5.50	0.51	1.00	0.50	0.01	1.00	0.08	0.03
6.00	0.50	1.00	0.50	0.01	1.00	0.06	0.01
**1.36**	**0.94**	**0.94**	**0.95**	**0.95**	**0.94**	**0.89**	**0.94**

Table S4. Performance of ThermoScan based on the different thresholds (TH) of average paragraph/table scores, ranging from 1 to 6. The performance measures are defined in the Methods section. The standard deviations of all performance measures are ≤0.02.

Words	TH	Q2	TNR	NPV	TPR	PPV	MCC	F1	AUC	AUPR
ThermoScan	3	0.97	1.00	0.95	0.94	1.00	0.94	0.97	0.99	0.99
TC	2	0.93	1.00	0.87	0.85	1.00	0.86	0.92	0.96	0.97
TV ∪ UM	3	0.89	0.93	0.86	0.85	0.92	0.78	0.89	0.96	0.96
TC ∪ TV ∪ UM	3	0.97	0.99	0.95	0.94	0.99	0.93	0.97	0.99	0.99

Table S5. Optimized performance of ThermoScan and 3 alternative methods on the training set (Pos-PT, Neg-PS and Neg-PU), based on the maximum paragraph/table score. TC: Thermodynamic Concepts. TV: Thermodynamic Variables. UM: Units of Measure. The performance measures are defined in the Methods section. The standard deviations of all performance measures are ≤0.01.

Words	TH	Q2	TNR	NPV	TPR	PPV	MCC	F1	AUC	AUPR
ThermoScan	1.36	0.94	0.94	0.95	0.95	0.94	0.89	0.94	0.98	0.99
TC	0.37	0.83	0.83	0.83	0.83	0.83	0.66	0.83	0.91	0.91
TV ∪ UM	1.03	0.81	0.88	0.78	0.75	0.87	0.64	0.80	0.79	0.77
TC ∪ TV ∪ UM	1.39	0.93	0.93	0.93	0.93	0.93	0.87	0.93	0.98	0.98

Table S6. Optimized performance of ThermoScan and 3 alternative methods on the training set (Pos-PT, Neg-PS and Neg-PU), based on the maximum paragraph/table score. TC: Thermodynamic Concepts. TV: Thermodynamic Variables. UM: Units of Measure. The performance measures are defined in the Methods section. The standard deviations of all performance measures are ≤0.01.

Words	TH	Q2	TNR	NPV	TPR	PPV	MCC	F1	AUC	AUPR
ThermoScan	3	0.80	0.49	0.88	0.96	0.78	0.55	0.86	0.86	0.86
TC	2	0.76	0.54	0.69	0.87	0.78	0.44	0.82	0.76	0.79
TV ∪ UM	3	0.71	0.71	0.57	0.72	0.82	0.41	0.77	0.81	0.80
TC ∪ TV∪ UM	3	0.79	0.47	0.87	0.96	0.78	0.53	0.86	0.85	0.84

Table S7. Performance of ThermoScan and 3 alternative methods on the New-PSU dataset, based on the maximum paragraph/table score. TC: Thermodynamic Concepts. TV: Thermodynamic Variables. UM: Units of Measure. The performance measures are defined in the Methods section.

Words	TH	Q2	TNR	NPV	TPR	PPV	MCC	F1	AUC	AUPR
ThermoScan	3	0.91	0.75	0.88	0.96	0.92	0.76	0.94	0.96	0.94
TC	2	0.82	0.68	0.64	0.87	0.89	0.54	0.88	0.83	0.83
TV ∪ UM	3	0.76	0.91	0.52	0.72	0.96	0.55	0.82	0.90	0.86
TC ∪ TV ∪ UM	3	0.90	0.72	0.87	0.96	0.91	0.73	0.94	0.95	0.93

Table S8. Performance of ThermoScan and 3 alternative methods on the Snew-PSU dataset, based on the maximum paragraph/table score. TC: Thermodynamic Concepts. TV: Thermodynamic Variables. UM: Units of Measure. The performance measures are defined in the Methods section.

Words	TH	Q2	TNR	NPV	TPR	PPV	MCC	F1	AUC	AUPR
ThermoScan	1.36	0.80	0.59	0.77	0.91	0.81	0.53	0.85	0.83	0.82
TC	0.37	0.65	0.10	0.46	0.94	0.66	0.07	0.78	0.56	0.56
TV ∪ UM	1.03	0.66	0.81	0.50	0.58	0.86	0.37	0.69	0.76	0.74
TC ∪ TV ∪ UM	1.39	0.79	0.59	0.76	0.90	0.81	0.53	0.85	0.80	0.80

Table S9. Performance of ThermoScan and 3 alternative methods on the New-PSU dataset, based on the maximum paragraph/table score. TC: Thermodynamic Concepts. TV: Thermodynamic Variables. UM: Units of Measure. The performance measures are defined in the Methods section.

Words	TH	Q2	TNR	NPV	TPR	PPV	MCC	F1	AUC	AUPR
ThermoScan	1.36	0.89	0.83	0.75	0.91	0.94	0.71	0.92	0.92	0.91
TC	0.37	0.73	0.11	0.37	0.94	0.76	0.08	0.84	0.59	0.57
TV ∪ UM	1.03	0.66	0.91	0.42	0.58	0.95	0.42	0.72	0.83	0.79
TC ∪ TV ∪ UM	1.39	0.87	0.79	0.73	0.90	0.93	0.67	0.91	0.90	0.89

Table S10. Performance of ThermoScan and 3 alternative methods on the Snew-PSU dataset, based on the maximum paragraph/table score. TC: Thermodynamic Concepts. TV: Thermodynamic Variables. UM: Units of Measure. The performance measures are defined in the Methods section.
